# Luhong Granules Prevent Ventricular Remodelling after Myocardial Infarction by Reducing the Metabolites TMAO and LPS of the Intestinal Flora

**DOI:** 10.1155/2019/8937427

**Published:** 2019-11-16

**Authors:** Tianshu Yang, Huiyan Qu, Xiaolong Song, Qian Liu, Xiaoli Yang, Jijie Xu, Tao Yang, Zhenzhen Lan, Wanjing Sha, Hua Zhou

**Affiliations:** Cardiovascular Research Institute and Cardiovascular Shuguang Hospital, Shanghai University of Traditional Chinese Medicine, Shanghai 201203, China

## Abstract

**Background:**

Ventricular remodelling is a common pathological change at all stages of heart disease. Luhong granules are widely used in patients with chronic ventricular remodelling after myocardial infarction and can alleviate chest tightness, shortness of breath, and other symptoms. However, its effect on ventricular remodelling remains to be studied.

**Purpose:**

In this study, we investigated the effects of these granules on myocardial fibrosis in a rat model of myocardial infarction in vivo.

**Methods:**

Male Wistar rats were randomly divided into four groups: the sham operation group, the acute myocardial infarction (AMI) group, the Luhong granule group, and the vancomycin group, with a sample size (*n*) of 10 rats in each group. The AMI model was established in all rats by ligation of the left anterior descending (LAD) coronary artery (the sham operation group did not undergo ligation). Luhong granules (0.5 ml·kg^−1^·d^−1^), vancomycin (0.075 g·ml^−1^·d^−1^), and 0.9% saline (5 ml·kg^−1^·d^−1^ for the sham operation and AMI groups) were administered orally for 6 weeks. Echocardiography was used to check cardiac structure and function. Myocardial and small intestinal tissue morphology was observed by haematoxylin and eosin (H&E) staining, and heart samples were stained with Masson's trichrome to analyse myocardial fibrosis. 16S rDNA sequencing was performed to detect changes in the gut flora. The level of trimethylamine N-oxide (TMAO) in plasma samples was quantified by stable isotope dilution liquid chromatography-tandem mass spectrometry (LC-MS).

**Results:**

H&E and Masson's trichrome staining of cardiac tissues showed that Luhong granules could partially reverse ventricular remodelling and improve intestinal barrier function (*P* < 0.05). Echocardiographic analysis showed that, compared with the AMI group, the left ventricular ejection fraction (LVEF) in the Luhong granule group was increased (*P* < 0.05). Stool sequencing and microbiological analysis showed changes in *Bacteroidales*, *Alistipes*, *Phascolarctobacterium*, etc., which can produce TMAO. We found that Luhong granules can reduce *Bacteroidales*, *Alistipes*, and *Phascolarctobacterium* at the genus level. The levels of TMAO and lipopolysaccharides (LPS) in plasma samples were reduced in the Luhong granule group (*P* < 0.05).

**Conclusions:**

Our results indicate that Luhong granules reduce TMAO and LPS levels in circulating blood by improving intestinal flora and intestinal barrier function to delay ventricular remodelling after myocardial infarction.

## 1. Introduction

Heart failure is developing into a modern epidemic, and despite advancements in treatment, heart failure still has ominous prognosis and results in major socioeconomic burden [1]. Although many new drugs have emerged as promising agents against heart failure, they have failed to improve residual morbidity and mortality [2, 3]. The key pathological mechanism of the occurrence and development of heart failure is ventricular remodelling. Myocardial fibrosis is an important pathological process in which ventricular remodelling occurs. The important role of the gut microbiota in the pathogenesis of cardiovascular diseases has been demonstrated [4]. Previous studies have proposed the role of the intestinal microbiota in cardiac fibrosis [5]. Trimethylamine N-oxide (TMAO), a gut microbiota-derived metabolite, dietary choline, and other trimethylamine containing nutrients are highly associated with cardiovascular risk [6]. Dietary choline and carnitine can be metabolized to trimethylamine (TMA) by the intestinal microbiota. Subsequently, TMA is absorbed through the portal vein and rapidly converted to TMAO by enzymes of the flavin monooxygenase (FMO) family, especially FMO3 and FMO1, in the liver [7]. In recent years, TMAO has been shown to be a novel independent risk factor for major adverse cardiovascular events in patients with heart failure [8]. Thus far, it is unclear how to treat structural and functional damage to the heart caused by high TMAO levels. Until now, no drugs have been found to treat myocardial fibrosis after myocardial infarction. The discovery of better treatments to reduce cardiac remodelling after myocardial infarction has become a new target for researchers. Thus, there is an urgent need to identify alternative treatments to attenuate ventricular remodelling after myocardial infarction. In recent years, researchers have attached great importance to the application of traditional Chinese medicine in the treatment of various diseases [9]. Luhong granules are mainly used for patients with chronic heart failure, especially patients who suffer from a heart attack. Because of its long history and continued use in most Chinese patients, we studied the mechanism of Luhong granules in preventing ventricular remodelling after myocardial infarction.

## 2. Materials and Methods

### 2.1. Animals

All study protocols were approved by the Animal Care and Use Committee of the Shanghai University of Traditional Chinese Medicine and were in strict accordance with the guidelines of the National Institutes of Health for Laboratory Animals (NIH publication No. 85–23, revised 1996). Adult male Wistar rats (weighing 200–220 g) were obtained from Beijing Vital River Laboratory Animal Technology Co., Ltd. (*n* = 10 for each group). All animals were reared in a temperature-controlled (20 ± 5°C) and a humidity-controlled (40–70%) environment. Animal facilities adhered to a 12 h light/dark cycle. The rats were given water and rodent chow ad libitum (supplied by the Shanghai University of Traditional Chinese Medicine Experimental Animal Center, China).

### 2.2. Drugs and Reagents

All crude ingredients for Luhong granules were purchased from Shanghai Hua-Yu Chinese Herbs Co., Ltd. (Shanghai, China). The Luhong granules consisted of 30 g of *Astragalus membranaceus* (Fisch.) Bge. var. mongholicus (Bge.) Hsiao (lot# 20121209) (*in TCM, the root of Astragalus membranaceus is used as crude drug*), 30 g of *Codonopsis pilosula* (Franch.) Nannf. (lot# 20110503) (*in TCM, the root of Codonopsis pilosula is used as crude drug*), 20 g of *Lepidium apetalum* Willd (lot# 20111004) (*in TCM, the mature seed of Lepidium apetalum Willd is used as crude drug*), 9 g of *Cervus nippon* Temminck (lot# 20121022) (*in TCM, the corner block for antlers of Cervus nippon Temminck is used as crude drug. In the spring and autumn seasons, the ossification corners are removed to the gums, and the corner pieces are taken out and dried*), 9 g of *Carthamus tinctorius* L. (lot# 20120603) (*in TCM, the dry flower of Carthamus tinctorius L. is used as crude drug*), and 9 g of *Cinnamomum cassia* Presl (lot# 20120712) (*in TCM, the dry branch of Cinnamomum cassia Presl is used as crude drug*). For quality control, Luhong granules were analysed by a UHPLC-Q Exactive System (Thermo, San Jose, CA, USA) [10]. Vancomycin was purchased from Servier, Shanghai Source Liquid Biotechnology Co., Ltd. (1404-93-9).

### 2.3. Animal Models

Rats were anaesthetized with an intraperitoneal injection of 1.5% sodium pentobarbital (Sigma-Aldrich; Merck KGaA) at a dose of 30 mg/kg body weight. Left intercostal thoracotomy was performed to expose the heart, and the left anterior descending (LAD) coronary artery was ligated with polypropylene suture under controlled ventilation [11]. Successful induction of acute myocardial infarction (AMI) was observed by the operators when the anterior and apical left ventricular myocardium gradually attained a dull, pale colour with reduced pulse. At the same time, electrocardiogram exhibited a gradual ST segment elevation in Lead II. Rats in the sham operation group underwent thoracotomy and pericardiotomy without LAD artery ligation. Rats that underwent MI surgery were randomly divided into three groups: the AMI group (0.9% saline), the Luhong granule group (0.5 ml·kg^−1^·d^−1^), and the vancomycin group (0.075 g·ml^−1^·d^−1^).

### 2.4. Design and Allocation of Rats

Rats were randomly divided into 4 groups: the sham operation group, the AMI group, the Luhong granule group, and the vancomycin group, with a sample size (*n*) of 10 rats in each group. Rats in the Luhong granule group were administered oral doses of Luhong granules at 0.5 ml·kg^−1^·d^−1^. Rats in the vancomycin group were treated with 0.075 g·ml^−1^·d^−1^ of vancomycin. Luhong granules and vancomycin were mixed with distilled water prior to administration. Rats in the sham operation group and the AMI group were administered equivalent amounts of 0.9% saline orally each day.

Starting from the day after LAD artery ligation, all rats were treated for 6 weeks. For 6 weeks, the postoperative rats were given a chow diet in the absence or presence of antibiotics or Luhong granules, and 0.9% saline was given to sham-operated rats. At the end of the treatment period, all rats were weighed.

The animals were fasted for 8 hours, and then blood and tissue samples were collected for further analysis. All rats were sacrificed by pentobarbital anaesthesia (0.04 ml/10 g, intraperitoneal injection). The heart and small intestine were quickly removed and stored in liquid nitrogen or 4% paraformaldehyde for further use.

### 2.5. Echocardiographic Assessment

At 6 weeks after the coronary artery occlusion surgery, all groups of rats were weighed and anaesthetized with 1.5% sodium pentobarbital. Echocardiography was performed on a multifunctional ultrasound system by noninvasive transthoracic echocardiography (Netherlands Philips IE33). Cardiac function was assessed using the left ventricular ejection fraction (LVEF). Each echocardiogram value was taken from the average of three consecutive cardiac cycles. Echocardiography was performed by a technician who was blinded to the assignment of groups.

### 2.6. Pathological Evaluation of Myocardial and Small Intestinal Tissues

Fixed hearts were laterally cut along the central plane to create a cross section on the left and right sides of the right ventricle. Fixed small intestines were sagittally sectioned. Heart and small intestinal tissues were embedded in paraffin and sliced (5 mm thick). Myocardial morphology was detected by haematoxylin and eosin (H&E) staining. Heart samples were stained with Masson's trichrome to analyse myocardial fibrosis. Images were then captured using a Leica Microsystems microscope and quantified using Image-Pro Plus 6.0.

### 2.7. Quantification of TMAO and LPS

The level of TMAO in plasma samples was quantified by stable isotope dilution liquid chromatography-tandem mass spectrometry (LC-MS). The internal standard trimethylamine-d9 N-oxide TMAO (d9-TMAO) was added to the serum sample prior to protein precipitation and monitored in the positive MRM mode (m/z 85 ⟶ 66). A serum analyte quantitative calibration curve was prepared by adding TMAO standards and a fixed amount of internal standard to the control serum. The level of lipopolysaccharides (LPS) in plasma samples was quantified using a fully automatic bioanalysis machine (Abbott Company, AxSYM, USA). Both technical support and equipment were provided by Shuguang Hospital Affiliated to Shanghai University of Traditional Chinese Medicine of Shanghai.

### 2.8. Stool Sampling, Sequencing, and Microbiological Analysis

DNA extraction and PCR amplification: immediately after euthanasia, rectal faecal specimens were placed in sterile tubes and stored at −80°C until treatment. Total DNA extraction was performed according to the instructions of the E.Z.N.A.® soil kit (Omega Bio-Tek, Norcross, GA, USA). DNA concentration and purity were measured using NanoDrop 2000, and DNA extraction quality was measured by 1% agarose gel electrophoresis. We used 338F (5′-ACTCCTACGGGAGGCAGCAG-3′) and 806R (5′-GGACTACHVGGGTWTCTAAT-3′) primers for the PCR amplification of the V3-V4 variable region. The amplification procedure was as follows: predenaturation at 95°C for 3 min, 27 cycles (denaturation at 95°C for 30 s, annealing at 55°C for 30 s, and extension at 72°C for 30 s), and extension at 72°C for 10 min (PCR: ABI GeneAmp® 9700). The amplification volume was 20 *μ*l, comprising 4 *μ*l 5 ∗ FastPfu buffer solution, 2 *μ*l 2.5 mM dNTPs, 0.8 *μ*l primer (5 *μ*M), 0.4 *μ*l FastPfu polymerase, and 10 ng DNA template.

Illumina MiSeq: PCR products were recovered using a 2% agarose gel using an AxyPrep DNA Gel Extraction Kit (Axygen Biosciences, Union City, CA, USA).

Purification, Tris-HCl elution, and 2% agarose electrophoresis: QuantiFluor™-ST (Promega, USA) was used for assay quantification. According to the Illumina MiSeq platform (Illumina, San Diego, USA) and standard operating procedures, the purified amplified fragments were constructed into a library of PE 2 ∗ 300.

### 2.9. Statistical Analysis

All statistical analyses were performed using SPSS software (version 21). Data were expressed as the mean ± standard deviation (*x* ± *s*). Data were analysed by homogeneity of variances and one-way analysis of variance (ANOVA). The least significant difference (LSD) method was used for individual comparisons. A value of *P* < 0.05 was considered significant.

## 3. Results

### 3.1. Luhong Granules Prevent Ventricular Remodelling after Myocardial Infarction

**Figure 1:**
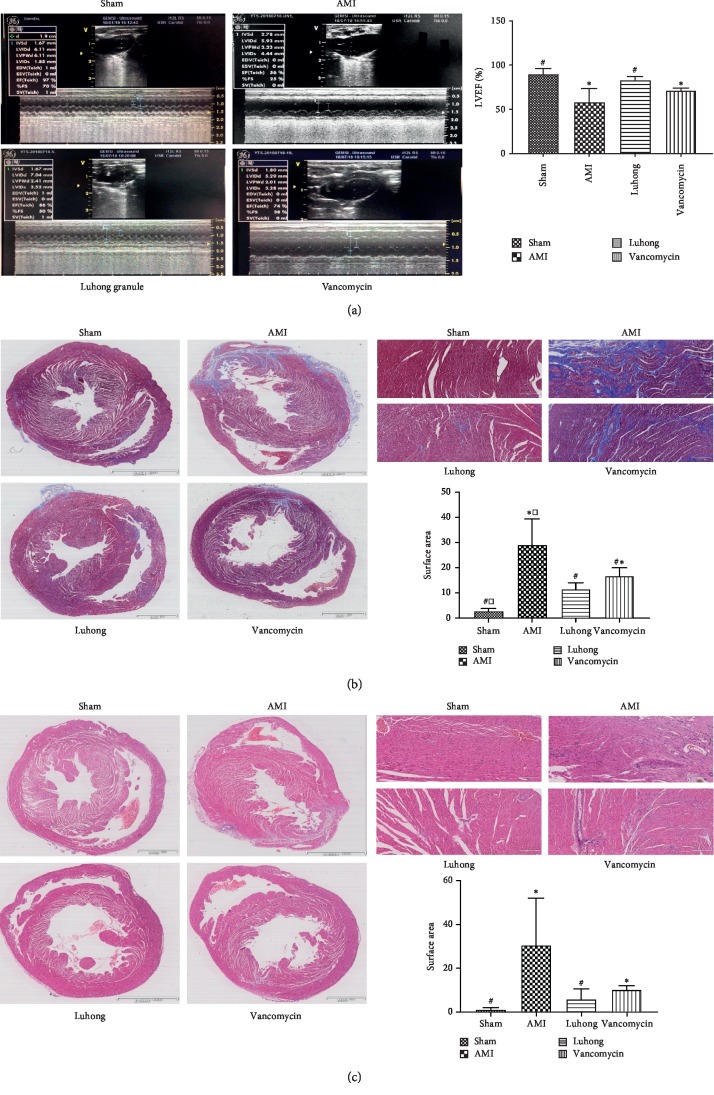
Luhong granules prevent ventricular remodelling after myocardial infarction: (a) echocardiographic analysis of AMI indicated exacerbated left ventricular dysfunction (represented by LVEF) in rats; (b) Masson's trichrome staining of heart tissues to examine rat cardiac fibrosis; (c) H&E staining of heart tissues to examine rat myocardial tissue necrosis vs sham, ^*∗*^*P* < 0.05; vs AMI, ^#^*P* < 0.05; vs vancomycin, ^□^*P* < 0.05.

In vivo, cardiac ultrasound can be used to directly assess cardiac function. AMI exacerbates left ventricular dysfunction (indicated by LVEF) in rats. Echocardiographic analysis showed that Luhong granules inhibited ventricular remodelling and preserved cardiac function, as shown by an increase in LVEF (Figure 1(a)). Compared with the sham operation group, the LVEF in the AMI group was reduced (*P* < 0.05). Compared with the AMI group, the LVEF in the Luhong granule group and the vancomycin group was increased (*P* < 0.05).

Ventricular remodelling is usually accompanied by fibrosis. Next, we performed H&E and Masson's trichrome staining on heart tissues to examine the effect of Luhong granules on rat heart fibrosis (Figures 1(b) and 1(c)). H&E and Masson's trichrome staining of cardiac tissues showed that, in the sham operation group, the colour of the myocardium was bright red, and the cells were in good condition and neatly arranged. In comparison with the sham operation group, the AMI group displayed a large amount of myocardial tissue necrosis, myocardial cell disorders, and increased cellular gap. However, inflammatory cell infiltration, local myocardial fibre rupture, and atrophy and degeneration of interstitial fibrous tissues were improved in the Luhong granule group and the vancomycin group. Masson trichrome staining showed significantly increased heart fibrosis in rats in the AMI group compared with the sham-operated rats, which could be prevented by vancomycin and Luhong granule treatment.

Notably, the suppression of intestinal barrier permeability, reduction in LPS and TMAO levels, preservation of cardiac function, and slowing of myocardial fibrosis and inflammation were not significantly different between the Luhong granule group and the vancomycin group. However, the Luhong granule group was superior to the vancomycin group in terms of symptom alleviation (hair colour change and asthma) in rats.

### 3.2. Luhong Granules Change the Intestinal Mucosal Barrier of Rats after AMI

Since the integrity of the intestinal barrier is closely related to changes in the gut microbiota and LPS levels [12–14], we further evaluated the changes in the intestinal barrier and LPS levels after treatment with Luhong granules.

**Figure 2:**
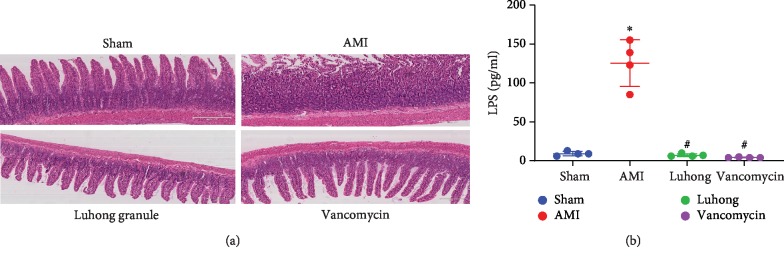
Effect of Luhong granules on gut barrier and serum LPS levels: (a) histopathological examination of rat distal ileum (H&E) showed mucosal injury at 6 weeks after AMI; (b) plasma levels of LPS in a rat model were measured at 6 weeks after AMI; vs sham, ^*∗*^*P* < 0.05; vs AMI, ^#^*P* < 0.05.

At 6 weeks after AMI, histological measurements of ileal morphology showed significant effects of the interventions on the intestinal tissues (Figure 2(a)). The sham operation group displayed tightly arranged and neatly ordered intestinal villi. However, the Luhong granule and vancomycin groups had larger intestinal villus clearance than the sham operation group, but the villi were arranged neatly. Compared with the above three groups, the AMI group had rough, dull, and irregularly arranged microvilli, with partial loss of intestinal epithelial cells.

Compared with the sham operation group, the serum LPS level in the AMI group was increased (*P* < 0.05). Compared with the AMI group, the serum LPS level in the vancomycin group and the Luhong granule group was decreased (*P* < 0.05) (Figure 2(b)). Therefore, Luhong granules could significantly prevent intestinal barrier damage and endotoxin (LPS) release into the circulation.

### 3.3. Luhong Granules Can Significantly Reduce TMAO Levels in Rats

**Figure 3:**
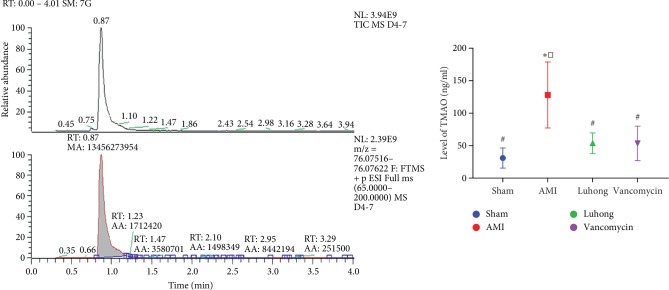
Plasma levels of TMAO were reduced by Luhong granules in a rat model at 6 weeks after AMI. The level of TMAO in plasma samples was quantified by stable isotope dilution LC-MS. vs sham, ^*∗*^*P* < 0.05; vs AMI, ^#^*P* < 0.05; vs vancomycin, ^□^*P* < 0.05.

Next, we investigated whether changes in the gut flora and intestinal pathology were associated with TMAO levels in circulating blood. The level of TMAO in plasma samples was quantified by stable isotope dilution LC-MS. As shown in Figure 3, compared with the sham operation group, the serum TMAO level in the AMI group was increased (*P* < 0.05). In addition, compared with the AMI group, the serum TMAO level in the vancomycin group and the Luhong granule group was decreased (*P* < 0.05). In conclusion, Luhong granule-mediated reduction in the plasma levels of TMAO may be due to regulation of the intestinal flora and prevention of increased intestinal permeability.

### 3.4. Effect of Luhong Granules on Intestinal Microbial Composition in Rats after AMI

We performed 16S rDNA sequencing of collected stool samples from rats in the AMI and sham operation groups approximately 6 weeks after surgery. After quality control, a total of 705,500 sequences were obtained, and all sequences were divided into operational taxonomic units (OTUs) according to different similarity levels. A total of 780 different OTU subgroups were identified, and each OTU was statistically analysed at 97% of the similarity level.

**Figure 4:**
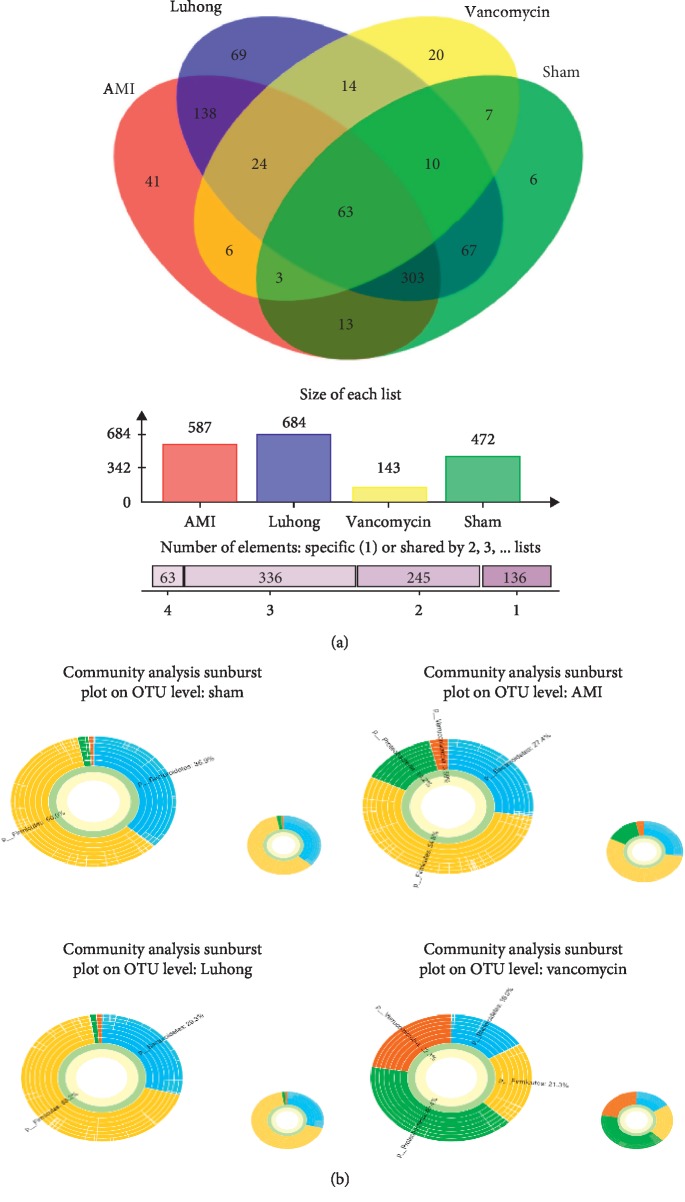
Microbial populations in the faeces of rats treated with Luhong granules at 6-week after AMI. Oral administration of Luhong granules (0.75 ml/kg/day) and vancomycin (0.075 g/kg/day) changed the total microbial numbers and altered the abundance of microbial taxa. (a) Venn diagram: the Luhong granule group (684) had the highest species richness, followed by the AMI (587) and sham operation (472) groups, while the vancomycin group (143) had the lowest species richness. (b) Sunburst diagram: at the genus level, *Firmicutes* and *Bacteroides* were the two most important genera in the sham operation, AMI, and Luhong granule groups. *Proteobacteria*, *Verrucomicrobia*, *Firmicutes*, and *Bacteroides* are the main genera in the vancomycin group.

Based on species composition analysis at the OTU level in faecal samples from the four groups, as shown by the Venn diagram, the abundance of species in the AMI group was significantly higher than that in the sham operation group. The Luhong granule group (684) had the highest species richness, followed by the AMI (587) and sham operation (472) groups, while the vancomycin group (143) had the lowest species richness (Figure 4(a)). As shown in the multilevel species sunburst diagram, *Firmicutes* and *Bacteroides* were the two most important species in the sham operation, AMI, and Luhong granule groups because vancomycin administered orally changed total microbial numbers and altered the abundance of microbial taxa, which had *Proteobacteria*, *Verrucomicrobia*, *Firmicutes*, and *Bacteroides* as the main genera (Figure 4(b)). The relative abundance of *Firmicutes* and *Bacteroidetes* was not significantly different between the sham operation group and the AMI group at the same time point (*P* < 0.05), indicating the lack of significant change in *Firmicutes* and *Bacteroidetes* at the genus level after myocardial infarction.

### 3.5. Luhong Granules Reduce Kurtosis of the Intestinal Flora Associated with TMAO

**Figure 5:**
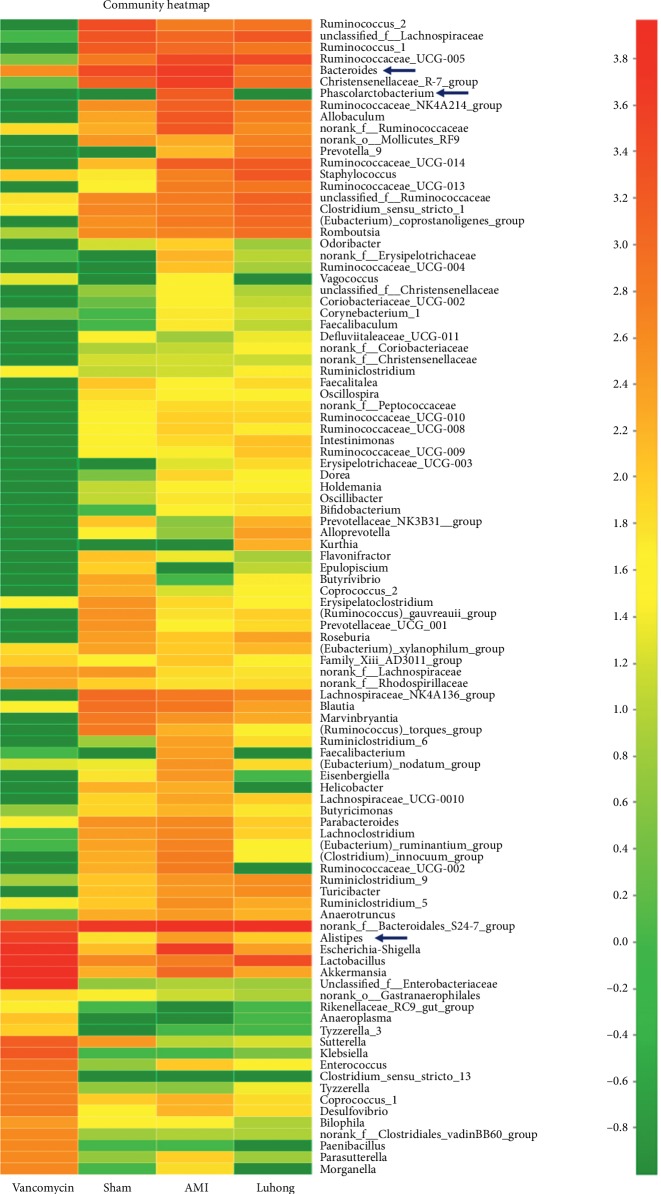
Luhong granules reduce kurtosis of the intestinal flora associated with TMAO, at the genus level.

As shown in the community heatmap, a certain colour gradient indicates the proportion of the species, and the right side of the figure represents the value denoted by the colour gradient. Compared with the AMI group, *Desulfovibrionaceae*, *Escherichia*, *Akkermansia*, *Bacteroidales*, *Alistipes*, and *Phascolarctobacterium* in the Luhong granule group and the sham operation group were significantly reduced (Figure 5). According to previous reports [15], *Clostridiales*, *Phascolarctobacterium*, *Oscillibacter*, *Bacteroidales*, and *Alistipes* promote TMAO generation. In this study, we found that Luhong granules can reduce the abundance of three genera that promote TMAO production, including *Bacteroidales*, *Alistipes*, and *Phascolarctobacterium* at the genus level. In addition, the abundance of bacteria was low in the vancomycin group, and the distribution of the flora was disrupted. These results indicate that Luhong granules can reduce TMAO content by changing the intestinal bacteria associated with TMAO. Moreover, long-term application of antibiotics leads to dysbacteriosis.

As shown in the community heatmap, the ordinate denotes the name of the species, and colour gradient represents the abundance of the species. The right side of the figure represents the value denoted by the colour gradient.

## 4. Discussion

In this study, we demonstrated that Luhong granules prevent ventricular remodelling after myocardial infarction by reducing the metabolites TMAO and LPS of the intestinal flora.

First, Luhong granules can reduce the abundance of intestinal bacteria that are positively associated with TMAO, such as *Bacteroidales*, *Alistipes,* and *Phascolarctobacterium*, at the genus level [15]. Second, treatment with Luhong granules reduces intestinal pathological changes. In addition, the levels of LPS and TMAO in the circulation were reduced after treatment with Luhong granules. Finally, as shown by echocardiography and myocardial pathology, Luhong granules protect the cardiac function of rats with myocardial infarction, prevent myocardial fibrosis, and finally delay ventricular remodelling after myocardial infarction. In summary, these data indicate that Luhong granules affect the intestinal flora and its metabolites TMAO and LPS, protecting cardiac function and delaying ventricular remodelling. Thus, we provide new insights into the pathological mechanism underlying ventricular remodelling after myocardial infarction.

The intestinal flora plays an important role in extracting energy from food and controlling local and systemic immunity. Current research reveals interesting findings of intestinal microbial regulation of development of cardiovascular diseases, and these data extend our understanding of the relationship between TMAO and cardiovascular risk [6]. Previous studies have shown that TMAO aggravates myocardial fibrosis [16]. However, other studies have shown that TMAO exerts a protective effect against myocardial fibrosis [17]. This difference can be explained by studying the differences in design, clinical background, geographic and ethnic backgrounds, or eating habits. Therefore, we designed this study to verify whether reducing the levels of TMAO and LPS in blood can improve ventricular remodelling after myocardial infarction. The increase in TMAO levels may be due to an increase in the average metabolic capacity of the intestine and a decrease in renal clearance. The intestinal hypothesis about the relationship between heart failure and the gut microbiota is that a large number of haemodynamic changes affect the growth and composition of the gut microbiota and promote TMAO and LPS translocation into the circulatory system, aggravating heart failure. To investigate whether increasing intestinal permeability can increase the levels of TMAO and LPS in circulating blood, we analysed the permeability of the small intestine and quantified the levels of TMAO and LPS in blood. Therefore, we hypothesized that altering the intestinal flora and intestinal permeability and reducing TMAO and LPS levels in blood can delay ventricular remodelling after myocardial infarction.

We established an AMI model, a well-characterized animal model of myocardial fibrosis. Myocardial infarction was induced according to a procedure previously described in the literature and adopted in our laboratory [18]. We observed that the induction of myocardial infarction increased the levels of TMAO and LPS, ultimately leading to myocardial fibrosis. Our LC-MS and fully automatic bioanalysis results showed that the levels of TMAO and LPS were increased in the myocardial infarction model, demonstrating the aggravation of myocardial fibrosis.

Luhong granules comprise *Astragalus membranaceus* (Huang Qi), *Codonopisis pilosula* (Dang Shen), *Lepidium apetalum* Willd (Ting Lizi), *Cervus nippon* Temminck (Lu Jiao), *Carthamus tinctorius* L. (Hong Hua), and *Cinnamomum cassia* Presl (Gui Zhi), which results in its function against ventricular remodelling after myocardial infarction by multicompound reactions [10], but the underlying mechanism remains unclear. In this study, we examined the effect of Luhong granules against ventricular remodelling. Ventricular remodelling after myocardial infarction is usually accompanied by complex intestinal changes. The intestinal abundance of *Clostridiales*, *Phascolarctobacterium*, *Oscillibacter*, *Bacteroidales,* and *Alistipes* is positively correlated with TMAO levels [15]. The flora correlated with TMAO can be detected in this study including *Bacteroidales*, *Alistipes*, and *Phascolarctobacterium*. Compared with the AMI group, the sham operation group and the Luhong granule group displayed significantly reduced abundance of the three genera. Luhong granules could significantly change the intestinal mucosal barrier. We also verified that Luhong granules reduce TMAO and LPS levels in circulating blood by improving the intestinal flora and intestinal barrier function. In addition, Luhong granules were not inferior to vancomycin in reducing TMAO and LPS.

In addition, in this study, cardiac ultrasound and pathological examination were performed in rats with myocardial infarction. The results showed that Luhong granules could significantly improve cardiac function in rats after myocardial infarction and slow the process of ventricular remodelling, and its curative effect is better than that of vancomycin. In the treatment of cardiac inflammation or fibrosis, the time and mode of administration are crucial. Vancomycin treatment in rats with ischaemia-reperfusion injury can reduce myocardial infarct size, but the direct injection of vancomycin into the coronary artery does not produce this effect [11]. The administration of antibiotics at initial stages of myocardial infarction to reduce myocardial inflammation can produce better results. Nonetheless, over time, the body becomes resistant to antibiotics. Therefore, widespread immunosuppression in late stages of heart failure is not useful for improving cardiac function. Since we did not examine late stages of heart failure, we observed no resistance to vancomycin in the rats.

In summary, our study demonstrates that Luhong granules prevent ventricular remodelling after myocardial infarction by reducing the metabolites TMAO and LPS of the intestinal flora. Several limitations include high cost and nonexistent consistency within the disease model. The application of Chinese medicine reflects advantages of simplicity and efficiency. Therefore, Luhong granules should be used as a carrier to explore the mechanism of improving ventricular remodelling after myocardial infarction.

## 5. Conclusions

In summary, our study showed that Luhong granules prevent ventricular remodelling after myocardial infarction by reducing the metabolites TMAO and LPS of the intestinal flora. Overall, Luhong granules may be a new agent for the treatment of myocardial fibrosis. Our findings demonstrate the potential of long-term clinical application of traditional Chinese medicine for treatment after myocardial infarction. In addition, our data contribute to our understanding of the effects of intestinal flora metabolites in ventricular remodelling and highlight the need to conduct more research in this field.

## Data Availability

The data used to support the findings of this study are available from the corresponding author upon request.
